# LOW-INTENSITY EXERCISE ENHANCES MUSCULAR ANDROGEN/ANDROGEN RECEPTOR TO INHIBIT MYOSTATIN PATHWAY

**DOI:** 10.1093/geroni/igz038.329

**Published:** 2019-11-08

**Authors:** Bo-Kyung Son, Masato Eto, Miya Oura, Masahiro Akishita

**Affiliations:** Department of Geriatric Medicine, Graduate School of Medicine, the University of Tokyo, Tokyo, Japan

## Abstract

Background: Physical exercise is well documented to induce muscle size, strength, and energy metabolism. Although the contribution of systemic or local androgen in exercise-adapted muscle hypertrophy has been suggested, less is known about the molecular pathway of androgen in response to exercise. In the present study, we examined roles of androgen/androgen receptor (AR) after exercise, especially for the suppression of myostatin, a potent negative regulator of muscle mass. Methods and Results: To examine the effects of exercise, we employed low-intensity exercise in mice and electric pulse stimulation (EPS) in C2C12 myotubes. Both mRNA and protein levels of AR significantly increased in skeletal muscle of low-intensity exercised mice and C2C12 myotubes exposed to EPS. Production of testosterone and DHT from EPS-treated C2C12 myotubes was markedly increased. Of interest, we found that myostatin was clearly inhibited by EPS, and its inhibition was significantly abrogated by flutamide, a specific antagonist of AR. Furthermore, IL-6 and phospho-STAT3 (pSTAT3) expression, the downstream pathway of myostatin, were decreased by EPS and this was also reversed by flutamide. Similar downregulation of myostatin and IL-6 was seen in skeletal muscle of low-intensity exercised mice. Conclusion: Muscle AR expression and androgen production were increased by exercise and EPS treatment. As a mechanistical insight, it is suggested that AR inhibited myostatin expression transcriptionally, which downregulates IL-6/pSTAT3 pathway and thus contributes to the prevention of muscle degradation.

